# Sustainable Weed Management

**DOI:** 10.3390/plants12081673

**Published:** 2023-04-17

**Authors:** Alessia Restuccia, Aurelio Scavo

**Affiliations:** Department of Agriculture, Food and Environment (Di3A), University of Catania, 95123 Catania, Italy; a.restuccia@unict.it

## 1. Introduction

Weeds are the most important biological constraint determining yield losses for field crops. For this reason, after World War II, synthetic herbicides have been largely adopted in developed countries in order to enhance yields and reduce the costs of cultivation. Unfortunately, their irrational use has caused environmental pollution, the development of herbicide-resistant weeds and shifts in weed communities, thus making cropping systems herbicide-dependent. Hence, following the ‘Zero Hunger’ goal of the United Nations Sustainable Development Goals and the strategies of the European Commission ‘Green Deal’, a weed management system based on cultural, mechanical, physical, biological and ecological methods to prevent or reduce the use of synthetic herbicides has become of outstanding importance in agricultural systems. Different techniques (cover cropping, the use of high-competitive cultivars, the choice of plant arrangement and seeding time, tillage systems, allelochemicals, etc.) and methodological approaches (e.g., soil seedbank analysis, weed adaptation along environmental gradients, and the analysis of weed abundance and diversity) have shown effectiveness in managing weeds from an eco-friendly perspective. The current Special Issue, entitled ‘Sustainable Weed Management’, was born within this context. It is a compilation of eighteen papers, including a review article related to the recent advancements in sustainable weed control methods and to biotic and abiotic factors affecting weed adaptation. The main topics covered by the Special Issue are:

- The effects of weed control practices on weed density and diversity;
- Cultural methods;
- Cover cropping and mulching;
- The use of allelopathic plant extracts and allelochemicals;
- Innovative chemical weeding methods.

## 2. Description of the Special Issue Main Findings

### 2.1. Weed Adaptation and Assemblages

Prior to analyzing the latest advancements in the wide area of weed control practices, nowadays, weed scientists are faced with the indirect effects of climate change on weed adaptation. Climate warming is inducing a high phenotypic plasticity in several weed species that may facilitate their invasive ability along environmental gradients. For this reason, Gentili et al. [1] used the seeds of the annual plant invader common ragweed (*Ambrosia artemisiifolia* L.) to determine variation in phenology and bio-morphological traits when grown along a 1000 m altitudinal gradient in Northern Italy, and under different temperature conditions in the growth chamber. They found that common ragweed may shift toward higher elevations and, at the same time, may improve the in situ (pre)adaptation of populations currently abundant at low elevations in the invasive European range. Another central topic in weed science is the determination of the processes that shape weed assemblages in farmlands. Studying the effects of crop competition on weeds, nitrogen input, weed control and landscape on both weed diversity and abundance in the margins and centers of 115 oilseed rape (*Brassica napus* L.) fields in Western France, Berquer et al. [2] found that landscape is the main driver of weed assemblages in field margins. In particular, they indicated crop height (i.e., competition) as the main driver for weed assemblages in field cores, and the number of meadows in the landscape (i.e., spatial dispersal) for weed assemblages in field margins.

### 2.2. Preventive Methods for Weed Management

In integrated weed management systems, indirect or preventive methods have a key role in reducing the impact and improving the effectiveness of direct control methods. Essentially, prevention is based on the management of the soil seedbank and an improvement in crop competitiveness against weeds. Preventive methods include crop rotation, cover cropping, mulching, the choice of row spacing and seeding rate, etc. Their combination is often associated with a higher weed-suppressive ability than a single method. For instance, the study by Naeem et al. [3] evaluated the impact of different weed management options (i.e., false seedbeds, allelopathic water extracts, chemical control, weed-free and weedy check) on weed flora in various barley-based cropping systems. From this study, it emerged that including mungbean (*Vigna radiata* (L.) R. Wilczek) or mainly sorghum (*Sorghum* spp.) in rotation with barley (*Hordeum vulgare* L.) and applying allelopathic water extracts could suppress weeds, similarly to herbicides. Hence, the combination of crop rotation and allelopathic water extracts was demonstrated as a valid alternative to herbicides in barley crop. Barroso and Genna [4] studied the effect of row spacing (18 or 36 cm) and seeding rate (73 or 140 kg ha^−1^) on Russian thistle (*Salsola tragus* L.) in spring barley and spring wheat crops in the Pacific Northwest. They concluded that increasing seeding rates or planting spring crops in narrow rows may be effective for yield increase in low-rainfall years of the zone under study, while no effect may be observed in years with higher rainfall than the normal trend. Concerning the role of highly competitive cultivars, Scavo et al. [5] conducted research over 10 farms in central–eastern Sicily on the weed-suppressive ability of old durum wheat landraces vs. modern cultivars in order to study the indirect effect of old landraces in sustainably reducing weed pressure without the adoption of chemical weed control. They reported that old durum wheat landraces were associated with a 47% reduction in the soil seedbank size and to a 64% decrease in the aboveground weed biomass compared to modern cultivars. Moreover, the weed species compositions of modern and old cultivars were quite separated for both soil seedbank and real flora, with the latter showing few specific associations with major weeds. The authors attributed the higher weed-suppressive ability of old durum wheat landraces to a combined competition–allelopathy effect.

### 2.3. Cover Cropping

Among the well-recognized ecosystem services provided by cover crops (i.e., non-harvested crops grown in addition to the primary cash crop with the aim of improving soil fertility and enhancing yields), the limitation of weeds is receiving more and more attention from the scientific community and stakeholders. Recently, Restuccia et al. [6] investigated the 5-year effect of subterranean clover (*Trifolium subterraneum* L.) and spontaneous flora, both with and without burying dead mulch into the soil, on weed abundance and diversity in a Mediterranean apricot orchard. They found that weed biomass was significantly reduced by subterranean clover, especially with burying dead mulch into the soil, with the cover crop biomass that was negatively correlated to weed biomass. Furthermore, compared to conventional apricot management, subterranean clover decreased the size of the soil seed bank by 57%. In a similar study, Las Casas et al. [7] studied the role of conservation agriculture and living mulches in a young Mediterranean olive orchard. The authors reported that the use of sage (*Salvia officinalis* L.) and lemongrass (*Cymbopogon citratus* (DC) Stapf) as living mulches combined to minimize soil disturbance, reduce the need for weed management, and promote the complexity of the Arthropod fauna in terms of both the number of species and the taxonomic complexity. Another technique related to cover cropping, i.e., mulching, was studied in this Special Issue by Ryan et al. [8] in winter wheat cultivated in central New York (USA). Evaluating a gradient of mulch biomass primarily composed of perennial species such as orchardgrass (*Dactylis glomerata* L.), timothy (*Phleum pratense* L.) and red clover (*T. pratense*), they found that wheat seedling density showed an asymptotic relationship with mulch biomass (no effect at low rates and a gradual decrease from moderate-to-high rates of mulch) and that the highest level of mulch (9000 kg ha^−1^) selectively suppressed weed biomass without reducing wheat grain yield.

### 2.4. New Advances in Chemical Weed Control

Herbicides still represent the most popular tool for weed control, mainly in developing countries. However, the study conducted by Pattanayak et al. [9] in an Indian sub-tropical environment highlighted that the chemical control with the herbicides bensulfuron, pretilachlor and bispyribac sodium negatively affected the soil microbial and enzymatic activity, whereas improved microbial populations and enzyme activities were noted in unpuddled transplanted rice (*Oryza sativa* L.) under organic weed management. Another negative effect related to the irrational application of herbicides is the spread of invasive or resistant weed species. Vázquez-García et al. [10] studied the resistance to acetyl-coenzyme A carboxylase (ACCase)-inhibiting herbicides in three resistant biotypes of *Phalaris*: *P. brachystachys*, *P. minor* and *P. paradoxa*. From their study, it emerged that cross-resistance in *Phalaris* species is conferred by specific point mutations, with *P. brachystachys* resistance that is due to target site and non-target-site resistance mechanisms, while only an altered target site was found in *P. minor* and *P. paradoxa*.

The present Special Issue pointed out different advances in the field of synthetic herbicides for the control of invasive and resistant weed biotypes. The use of tank-mix herbicides is one of these. For instance, Abu-Nassar and Matzrafi [11] indicated that tank mixes of oxadiazon and oxyfluorfen with different concentrations of surfactant significantly suppressed *Solanum rostratum* Dunal, an important invasive weed in Israel since the 1950s, when applied at a later growth stage (8–9 cm height). Additionally, Campos et al. [12] suggested a methyl-capped polyethylene glycol ester of pelargonic acid (PA-MPEG) in synergism with a non-phytotoxic alkylated seed oil-based adjuvant (i.e., Hasten^TM^) to improve the herbicidal efficacy of this novel fatty acid ester by disintegrating the bio-membranes and, thus, negatively affecting plant transpiration. O’Brien et al. [13] tested the effectiveness of a novel stem implantation system for controlling the woody weed Chinese elm (*Celtis sinensis* Pers.) in a conserved habitat. They found that the encapsulated glyphosate (245 mg/capsule), aminopyralid and metsulfuron-methyl (58.1 and 37.5 mg/capsule) and picloram (10 mg/capsule) achieved a similar herbicidal activity to the benchmark treatment (diesel + triclopyr + picloram + liquid hydrocarbon), because these encapsulated herbicides are immediately sealed into the vascular system of the target species, thus reducing the amount of active agent required and preventing environmental exposure.

### 2.5. Use of Allelopathy for Weed Management

Allelopathic species can be manipulated for the sustainable management of weeds in different ways such as the introduction of an allelopathic crop into crop rotation schemes [3], the use of an allelopathic cover crop [6], or the identification, isolation and extraction of plant allelochemicals for the possible production of bioherbicides. In this Special Issue, the bioherbicidal potential of the essential oils from Mediterranean Lamiaceae members was reviewed by De Mastro et al. [14]. In addition, Motmainna et al. [15] investigated the allelopathic potential of *Parthenium hysterophorus* L. methanolic extracts at different concentrations under laboratory and glasshouse conditions. They indicated eight amino acids, seven phenolic compounds, three terpenoids and other secondary organic compounds as *P. hysterophorus* allelochemicals in methanolic extract. The *P. hysterophorus* extract was also capable of inhibiting the germination and growth of *Cyperus iria* L. to a similar extent to the synthetic herbicides glyphosate and glufosinate-ammonium. Another study evaluated the thermal allelopathic effect of two coniferous plants (*Pinus densiflora* Siebold & Zucc. and *Pinus koraiensis* Siebold & Zucc.) on oilseed rape (*B. napus*) germination and seedling growth in order to assess whether high temperatures, generated during composting, decrease allelopathic ability [16]. It was found that the allelopathic capacity of two *Pinus* species showed root-specific inhibition, but the decrease in volatile contents after the thermal process was lesser in *P. koraiensis* than in *P. densiflora*. The authors, therefore, suggested the application of the two conifer needles as allelopathic compost to control the initial weed growth in horticultural crops thanks to their thermal stability and root-specific inhibition.

Seed meals obtained from allelopathic crops are another allelopathic tool and eco-friendly alternative to synthetic herbicides. Pytlarz and Gala-Czekaj [17] assessed the allelopathic activity of seed meals from *Fagopyrum esculentum* Moench, *Sinapis alba* L., *Phacelia tanacetifolia* Benth., *Lupinus luteus* L., *Raphanus sativus* var. *oleiformis* and *Ornithopus sativus* Brot., at 1 and 3% doses, on herbicide-susceptible and -resistant (to propoxycarbazone-sodium) rye brome (*Bromus secalinus* L.) biotypes in winter wheat. They reported crop- and dose-dependent results. In particular, (1) wheat emergence and initial growth were not affected by the seed meals from *F. esculentum*, *P. tanacetifolia*, and *R. sativus* at 1% concentration in the soil; (2) the phytotoxicity of these seed meals was at the same level as the herbicide or higher; (3) an increase in seed meal concentration is not recommended due to the reduction in wheat emergence.

Plants’ allelopathic potential is known to be influenced by genotype, partly due to the different concentration of allelochemicals. Following the return to local durum wheat landraces demanded by the market, Scavo et al. [18] conducted research on the allelopathic effects of the extracts from three durum wheat landraces (‘Timilia’, ‘Russello’ and ‘Perciasacchi’) and a modern variety (‘Mongibello’), obtained from three different plant parts (ears, stems and roots), on the weeds *Portulaca oleracea* L. and *Stellaria. media* (L.) Vill. It was found that old landraces (mainly ‘Timilia’ and ‘Russello’) showed a higher allelopathic activity and that ear extracts were the most active. These results confirmed in the laboratory the findings obtained by Scavo et al. [5] in open-field conditions.

## 3. Conclusions

This Special Issue involves a wide range of knowledge, methods and practices recently achieved for sustainable weed management. Altogether, the papers published here demonstrate that effective weed control can be performed not only with an indiscriminate use of herbicides, but also with proper chemical weed control and with other eco-friendly methods including allelopathy, cover cropping, tillage, etc. It also emerged that the combination of different methods often results in an improved weed-suppressive ability.

As Guest Editors, we acknowledge all the authors for their submissions to our Special Issue. We believe that this excellent research is a significant breakthrough for current science and will be made available to farmers and stakeholders.

## Data Availability

Not applicable.
